# A journey from the endothelium to the tumor tissue: distinct behavior between PEO-PCL micelles and polymersomes nanocarriers

**DOI:** 10.1080/10717544.2018.1510064

**Published:** 2018-10-12

**Authors:** Agathe Figarol, Laure Gibot, Muriel Golzio, Barbara Lonetti, Anne-Françoise Mingotaud, Marie-Pierre Rols

**Affiliations:** a Institut de Pharmacologie et Biologie Structurale, IPBS, Université de Toulouse, CNRS, UPS, Toulouse, France;; b Laboratoire des IMRCP, Université de Toulouse CNRS UMR, Toulouse, France

**Keywords:** Nanocarriers, polymers, self-assembly, drug delivery, translocation, endothelium, tumor

## Abstract

Polymeric nanocarriers must overcome several biological barriers to reach the vicinity of solid tumors and deliver their encapsulated drug. This study assessed the *in vitro* and *in vivo* passage through the blood vessel wall to tumors of two well-characterized polymeric nanocarriers: poly(ethyleneglycol-b-ε-caprolactone) micelles and polymersomes charged with a fluorescent membrane dye (DiO: 3,3'-dioctadecyloxacarbo-cyanine perchlorate). The internalization and translocation from endothelial (human primary endothelial cells HUVEC) to cancer cells (human tumor cell line HCT-116) was studied in conventional 2D monolayers, 3D tumor spheroids, or in an endothelium model based on transwell assay. Micelles induced a faster DiO internalization compared to polymersomes but the latter crossed the endothelial monolayer more easily. Both translocation rates were enhanced by the addition of a pro-inflammatory factor or in the presence of tumor cells. These results were confirmed by early *in vivo* experiments. Overall, this study pointed out the room for the improvement of polymeric nanocarriers design to avoid drug losses when crossing the blood vessel walls.

## Introduction

Conventional anti-cancer treatments can lead to heavy side-effects. The use of nanocarriers aims at overriding nonspecific toxicity by optimizing the delivery of therapeutic agents while increasing their efficiency. The on-site delivery of the encapsulated compounds must overcome numerous defenses from the human body: biodegradation, opsonization and excretion, physiological barriers, and cellular-based mechanisms of drug resistance. This is a partial explanation for the nanocarrier’s long and complex development and their low clinical translation ‘from bench to bed.’ Recently, Wilhelm et al. (2016) reviewed 10 years of research on nanomedicine and concluded that the essential nanocarriers’ weakness was a too low ratio of administered versus delivered dose. According to his systematic literature review, this ratio reaches only a median of 0.7% in solid tumors. This article has been moderated, notably, because the specific context of nanomedicine can hardly be compared to the small molecules drugs’ one (Torrice, 2016). However, its perspective on the field remains interesting. To reach the targeted tumor after intravenous injection, nanocarriers must cross the endothelium formed by endothelial cells. This semi-permeable and size-selective barrier, lining the interior surface of blood vessels, controls the transport of gases, nutrients, or other materials through para- or transcellular ways. Tight intercellular junctions limit the paracellular passage to particles smaller than 6 nm in the general health conditions (Komarova & Malik, 2010). Transcellular passage is restricted by endosome vesicular transport. Accordingly, nanocarriers from 10 to 100 nm should only cross the healthy endothelium in minimal quantity. However, the path to tumor cells might be facilitated thanks to the enhanced permeability and retention phenomenon (EPR). The EPR theory, first described by Matsumura & Maeda (1986), stated that tumor environment triggers an increase in the endothelium permeability. The EPR effect is a complex phenomenon linked to (1) the angiogenesis leading to hypervascularity and facilitating access to the tumor environment for nanocarriers, (2) a change in the endothelium architecture leading to a higher permeability of the blood vessels, and (3) an alteration of the lymphatic system limiting the clearance of the nanocarriers once they have reached the tumor (Dvorak et al., 1988; Baban & Seymour, 1998; Maeda, 2001).

The choice of the nanocarrier and its physicochemical properties has an utterly important impact on tumor targeting efficiency. Amphiphilic block copolymers consist in the covalent association of a hydrophilic and a hydrophobic polymer. In appropriate solvents, and under appropriate temperature and shear stress, they can self-assemble to form polymeric self-assemblies with hydrophobic and hydrophilic compartments. The hydrophilic and hydrophobic weight fractions of the chosen block co-polymer dictate different self-assemblies geometries. Although far from universal, a hydrophilic weight fraction over 50% tends to result in micelles, whereas a hydrophilic fraction from 25% to 45% favors polymersomes (Discher & Eisenberg, 2002; Liu et al., 2012; Grossen et al., 2017). With larger diameters, polymersomes, a polymeric analog of liposomes, have the perk of being able to encapsulate both hydrophilic and hydrophobic compounds. Simpler to apprehend, micelles have been widely studied, but polymersomes are considered as particularly relevant for nanomedicine due to their tunable and higher encapsulation capacity (Balasubramanian et al., 2016). Other morphologies such as elongated micelles or vesicles are also possible. All these biodegradable polymeric self-assemblies were described as offering a balanced relationship among long blood circulation, robust encapsulation, tunable chemistry, and flexible structure (Soppimath et al., 2001; Christian et al., 2009; Li et al., 2016). Poly(ethyleneoxide-b-ε-caprolactone) (PEO-PCL) vesicles are one of the most promising candidates (Grossen et al., 2017). Both polyethylene oxide (PEO, often also called polyethylene glycol (PEG)) and polycaprolactone (PCL) are considered as biocompatible and have been approved by health-care authorities such as the Food Drug Administration (FDA) for use in drug delivery (Sui et al., 2015) and PEO-PCL nanocarriers seem to trigger no cytotoxicity (Zhang et al., 2013; Gibot et al., 2014; Sui et al., 2015). There are, moreover, abundant publications on the use of a PEGylated surface to increase blood circulation time of nanocarriers by avoiding opsonization (Li et al., 2014; Owens III & Peppas, 2006). To reach a balance between low excretion rate and high encapsulation potency, Alexis et al. (2008) suggested an optimal size of PEGylated-nanocarriers between 10 and 100 nm and Grossen et al. (2017) between 6 and 500 nm. In this size range, PEO-PCL micelles (PEO-PCL 5000-4000 g.mol^−1^) and PEO-PCL polymersomes (PEO-PCL 5000-11000 g.mol^−1^) were chosen following previous studies of our laboratories confirming their efficiency as nanocarriers of pheophorbide A for photodynamic therapy (Gibot et al., 2014; Till et al., 2016a, 2016c).

The goal of this study was to compare the interaction of two nanocarriers’ (micelles and polymersomes) path from endothelium lining blood vessels to the tumor cells. In this study, to avoid cytotoxicity and for biological imaging convenience, the encapsulated agent was not a therapeutic one but the green fluorophore DiO (3,3′-dioctadecyloxacarbo-cyanine perchlorate). First, we assessed the carrier’s internalization by human endothelial cells (HUVEC) and determined if there is specificity in internalization between those cells and human colorectal carcinoma cells (HCT-116). We then assessed the nanocarriers’ ability to cross the endothelium in healthy conditions and in the vicinity of cancer cells to deliver its encapsulated compound. To do so, observations and quantification of internalization were first carried out on two-dimension (2D) conventional monolayer cell culture. Then, three-dimension (3D) HCT-116 spheroids served as model for tumor. HCT-116 spheroids have already been used as an acknowledged 3D tumor model reducing the gap between *in vitro* and *in vivo* outcomes (Chopinet et al., 2012; Gibot et al., 2014, 2013; Till et al., 2016b, 2016c). For the model of translocation across endothelium, an adaptation of transwell assays (on Boyden chambers) was used. Well-documented for chemotaxis, transwell assays only start to be used to study the passage of nanomaterials across biological barriers such as mucus, aortic, brain, and intestinal barriers (Broughton-Head et al., 2007; Jayagopal et al., 2008; Cohen et al., 2014; Shah et al., 2015; Kasper et al., 2016; Lin et al., 2016; Peuschel et al., 2016; Zhao et al., 2016; Boya et al., 2017; Hsiao et al., 2017). So far, this promising and flexible technique had not been used with polymeric nanocarriers or to mimic the endothelium. It handily provided information on *in vitro* behaviors of the PEO-PCL nanocarriers, before confirmation of the results by preliminary *in vivo* experiments.

## Materials and methods

### Chemicals

Poly(ethylene oxide-b-ε-caprolactone) with respective block molecular weights of 5000 and 4000 g.mol^−1^ (PEO-PCL 5-4), 5000 and 11000 g.mol^−1^ (PEO-PCL 5-11), and anthracene-terminated polycaprolactone (8100 g.mol^−1^) were obtained from Polymer Source Inc. (Dorval, Canada). 3,3'-Dioctadecyloxacarbo-cyanine perchlorate fluorophore (DiO) was purchased from Invitrogen Life Technologies (Saint Aubin, France). The solvents were purchased from SDS (Seihl, France) and unless specified otherwise, all other chemicals were purchased from Sigma-Aldrich (Saint-Quentin Fallavier, France).

### Nanocarrier formulations and characterizations

#### Nanocarrier formulations

Formulation of polymeric micelles by a co-solvent approach was carried out as previously described (Knop et al., 2009). Briefly, 20 mg of PEO-PCL 5-4 with or without DiO (2% mol) or PCL-Anthracene (10% mol) were dispersed in 0.4 mL acetone. This solution was added dropwise into 5 mL of ultrapure filtered water under continuous stirring. The resulting micelle suspension was left standing 48 h at room temperature for the acetone to evaporate. Polymersomes were formed by film rehydration-extrusion as previously described (Till et al., 2014, 2016b). A solution of 20 mg of PEO-PCL 5-11 in 1 mL chloroform with or without DiO (2% mol) or PCL-Anthracene (10% mol) was evaporated on a rotary evaporator. The resulting film was further dried under vacuum for at least 2 h. After rehydration in 2 mL of ultrapure filtered water, the sample was heated at 65 °C for 30 min without stirring, and 1 h in an ultrasonic bath. The sample was afterward extruded 10 times through a 0.4 µm cutoff polycarbonate membrane using a Polar Avanti Lipids mini-extruding system (Avanti Polar Lipids Inc., Alabaster, USA).

#### Nanocarrier physicochemical characterizations

The morphology of the polymeric micelles and polymersomes was checked using transmission electronic microscopy (TEM) on a Hitachi HT7700 microscope (Hitachi High-Technologies Corp., Tokyo, Japan). A copper grid coated with a carbon membrane was briefly immerged in a drop of micelles or polymersomes suspension. After removal of the excess of liquid on an absorbent paper, a few drops of a uranyl acetate solution were let for absorbance onto the grid for 10 s. The grid was finally dried for 3 min under a lamp before observations (acceleration voltage of 75 kV).

The nanocarriers hydrodynamic diameter was assessed using dynamic light scattering (DLS) on a Malvern Zetasizer Nano ZS (Malvern, Orsay, France). Samples were analyzed in triplicate undiluted at 25 °C with a 60 s record, standard laser at 633 nm and a 173° angle. Data were analyzed using the general-purpose non-negative least-squares method. The typical accuracy for these measurements was 10–20% for systems exhibiting a poly-dispersity index lower than 0.4. Further measurements were carried out on polymersomes to confirm their hydrodynamic diameters and polydispersity using Nanoparticle Tracking Analysis (NTA, NanoSight device from Malvern, Orsay, France). Samples were analyzed diluted at 1/10,000 at 25 °C with a 60 s record. Zeta potentials were measured on a Malvern Zetasizer Nano ZS at 25 °C with undiluted samples using Smoluchowski’s model. Nanocarriers were diluted at 2% v/v in ultrapure filtrated water before being analyzed using UV-vis (Specord S 600, Jena, Germany) from 200 to 800 nm at 37 °C. Dialyses of the nanocarriers were carried out to measure the DiO release over time such as in Guo’s study (Guo et al., 2017). Nanocarriers were diluted in ultrapure water to 1 µM of DiO. 2 mL of this suspension were dialyzed using mini Dialysis Kit at 1 kDa cutoff (GE, Pittsburgh, USA) against 500 mL of ultrapure water at 37 °C under slow stirring. Samples were analyzed by UV-vis at 0, 15, and 24 h. Further characterizations of PEO-PCL 5-11 morphology were performed using light and neutron scattering, as well as cryo-Transmission Electron Microscopy and are described in Supplementary information S2–S4.

### Internalization of nanocarriers by tumors and endothelial cells in conventional 2D monolayers and 3D tumor spheroids

Human colorectal carcinoma cells (HCT-116) were purchased from ATCC (#CCL-247). HCT-116 were grown in Dulbecco’s modified Eagles medium (DMEM) with 4.5 g.L^−1^ glucose, L-glutamine and pyruvate, supplemented with 10% (v/v) heat-inactivated fetal calf serum, 100 U.mL^−1^ penicillin and 100 μg.mL^−1^ streptomycin and used for experiments until passage 20. DMEM cell culture medium was purchased from Invitrogen Life Technologies (Saint Aubin, France), penicillin, streptomycin, and phosphate-buffered saline (PBS) were obtained from Sigma-Aldrich (Saint Quentin Fallavier, France). Primary human umbilical vein endothelial cells (HUVEC) were obtained from Lifeline Cell Technology (Frederick, USA) at passage 2 from a single donor. HUVEC were grown in EBM-2 medium complemented with the EGM-2 MV bullet kit without gentamycine, plus 100 U.mL^−1^ penicillin and 100 μg.mL^−1^ streptomycin and used for experiments until passage 6 in order to preserve their differentiated phenotype. EBM-2 medium and EGM-2 MV supplement kit were obtained from Lonza (Walkersvlle, USA). Cells were maintained at 37 °C in a humidified atmosphere containing 5% CO_2_.

Large aggregates of nanocarriers appeared in test conditions due to interaction between the polymeric nanocarriers and cell-treated plastic culture plates (five different brands tested). These aggregates have been avoided by the functionalization of cell culture plates with sterilized gelatin 0.4% in ultrapure water (see Supplementary data S1). Gelatin was purchased from Fisher Scientific (Loughborough, UK). A volume of gelatin equivalent to the planned volume of culture medium was added into the plates. They were then stored at 4 °C overnight before the gelatin was removed and the plates let dry for a few minutes under a laminar flow hood before use. Gelatin coating was chosen because of its high biocompatibility with cells.

First, cell viability was assessed by Prestoblue reagent (Invitrogen Life Technologies, Saint Aubin, France). Briefly, HCT-116 cells were seeded in 96-well plates at 30,000 cells/well, HUVEC at 15,000 cells/well and allowed growing for 24 h. Nanocarriers or DiO alone were then added (diluted at 50, 10, and 1 µM polymer and equivalent in DiO based on polymersomes content) for the toxicity assessment. After an incubation of 24 h, cells were then rinsed three times with PBS, 90 µL of culture medium and 10 µL of PrestoBlue were added. The plates were incubated at 37 °C, 5% CO_2_ for 30 min before absorbance reading at 570 nm and 600 nm on a spectrophotometer (Clariostar, BMG Labtech, Ortenberg, Germany).

DiO internalization was then visualized using confocal microscopy on classical 2D monolayer. HCT-116 and HUVEC were seeded in chambered coverglass previously functionalized with gelatin. After 24 h, cells were incubated with nanocarriers at 50 µM polymer (corresponding to 1 µM DiO) for an additional 24 h. Fresh cells were then rinsed three times with PBS and directly observed using confocal microscopy on a Fluoview FV1000 (Olympus, Rungis, France) after nuclei counterstaining with Hoechst, a fluorescent DNA intercalant. Laser wavelengths were 405 and 488 nm to observe Hoechst and DiO fluorescence, respectively. The 40× objective was used. Live cells were maintained at 37 °C under 5% CO_2_.

Kinetics of internalization of nanocarriers were analyzed on 2D monolayers by flow cytometry. 12-well plates were seeded with 40,000 cells/wells after 24 h cells were incubated with nanocarriers at 50 µM polymer for an additional 24 h. At different time points, cells were trypsinized and transferred to micro-cytometer tubes on ice. Cells were excited with a laser at 488 nm and DiO fluorescence was read with FL1 channel on a FACSalibur (BD Bioscience, Singapore, Singapore) with a total of at least 6000 cellular events collected for each tube.

In 2D co-culture, plasma membranes of HCT-116 cells were labeled with CellVue Claret Far Red Cell Linker according to the manufacturer protocol. Seeding density was optimized to obtain a 50–50% concentration in HCT-116 and HUVEC at the time of analysis (i.e. 48 h after seeding): 10,000 HCT-116 and 20,000 HUVEC/well in 12-well plates. After 24 h of cell growth, then 24 h of incubation with nanocarriers, DiO (488 nm excitation) and CellVue Claret (655 nm excitation) fluorescences were read on the FACSalibur on FL1 and FL4 channels, respectively, avoiding thus compensations.

HCT-116 spheroids were produced by the non-adherent techniques as previously described (Gibot et al., 2013). 5000 cells/well were seeded into gelatin-functionalized non-adherent U bottom 96 well plates (Costar #7007). Cells grew into spheroids for 5 days at 37 °C, 5% CO_2_. Spheroids were then harvested and introduced into new wells containing nanocarriers at 50 µM polymer in fresh medium. After 24 h of exposure, spheroids were rinsed twice in PBS and deposited on a chambered slide. DiO penetration in the spheroids was detected by a two-photon microscopy LSM 7MP device (Zeiss, Marly Le Roi Cedex, France) with a 20× objective, at room temperature. Chameleon Ultra 2 wavelength was set at 800 nm.

### In vitro *and* in vivo *nanocarriers translocation across the endothelium*

Transwells with post-confluent HUVEC monolayer were used to mimic the endothelium. Cells were seeded on 6.5 mm transwell inserts with 0.4 μm pore polyester membrane (Corning, New York, USA). In optimized conditions, 100,000 HUVEC were seeded on the top chamber (donor chamber) in 100 µL of culture medium, whereas the bottom chamber (receptor chamber) was filled with 600 µL of culture medium for 48 h of incubation at 37 °C, 5% CO_2_. The HUVEC monolayer was characterized using hematoxylin-eosin staining, scanning, and transmission electronic microscopy (SEM and TEM) and trans-endothelial resistance (TEER) measurement. After histological staining with Hematoxyling and Eosin colorant, membrane was cut out and mounted on a glass slide for imaging on a Leica MacroFluo device (Leica, Nanterre, France). TEM images were acquired on the Hitachi HT7700 microscope (Hitachi High-Technologies Corp., Tokyo, Japan), whereas SEM images were obtained with the Quanta 250 FEG (FEI, Oregon, USA). TEER was measured over time during the HUVEC monolayer formation with the EVOM-2 Volt-Ohm Meter and the ENDOHM-6 (WPI, Berlin, Germany). After 48 h of HUVEC monolayer formation on the inserts, the medium of the donor chamber was replaced by 100 µL of medium containing DiO-charged nanocarriers at a concentration of 50 µM of polymer. After another 24 h of incubation, the level of DiO in the receptor chamber was analyzed by fluorescence measurement at 488 nm on the fluorimeter Clariostar (BMG Labtech, Ortenberg, Germany). These levels of DiO were normalized to those of acellular controls. Nanocarriers both tagged with DiO (charged in the core of the micelles or polymersomes) and Anthracene (covalently linked to PCL) were used to distinguish the fate of the fluorophore and the polymers with a second fluorescence reading at 355 nm.

DiO passage was also assessed in an inflammatory context, to get closer to the context of the nanocarriers passage from blood vessels to tumor through altered endothelium. During the HUVEC monolayer formation on the inserts, at 24 h, the receptor chamber medium was replaced by medium with 600 µL of tumor necrosis factor α (TNF-α) at 100 U.mL^−1^. The experiment was then carried out as previously explained. Another step to enhance the endothelium model was to seed tumor cells in the bottom of the receptor well. 30,000 HCT-116 cells were thus seeded on the bottom of the 24-well plate just before plating HUVEC cells on the membrane above. Medium fluorescence was read with the fluorimeter, and cell fluorescence (for both HUVEC and HCT-116) was read with the flow cytometer.

*In vivo* early experiments were conducted to complete the *in vitro* findings. All animal experiments were conducted in agreement with the official guidelines of EU Directive 2010/63/EU and approved by the local ethics committee as a pilot study on small number of mice. Female BALB/c-nu mice were obtained from Janvier Labs (Saint Berthevin, France). Preliminary tests have shown that the DiO dose used *in vitro* (50 µM polymer were equivalent to 1 µM DiO) was too low to detect the fluorescence *in vivo*. Therefore, nanocarriers charged at 20% mol DiO instead of 2% mol DiO were formulated. Samples were diluted with PBS 10× to retrieve physiological osmolarity. Mice bearing sub-cutaneous established human HCT-116 tumors were treated with either control, DiO alone, PEO-PCL 5-4 micelles loaded with 20% mol DiO, or PEO-PCL 5-11 polymersomes loaded with 20% mol DiO. The concentration of DiO alone was equivalent to the DiO concentration in polymersomes, hence superior to the micelles one. A retro-orbital injection of 100 µL was carried out on one mouse per condition. At 24 h post-injection, all mice were sacrificed for further tissue analyses. Briefly, fluorescence of the whole organs of sacrificed mice was directly observed under Macrofluo microscope (Leica, Wetzlar, Germany) equipped with a cooled CCD camera (Roper Coolsnap HQ, Photometrics, Tucson, AZ) with a fluorescent filter at 488 nm, with a special focus on tumor tissue. Fluorescent pictures were obtained by use of CRI Micro*Color 2 Liquid Crystal Technology. Harvested tumors were fixed in formol (Sigma) for 24 h at 4 °C and then immersed for 2 h at 4 °C in 10% sucrose in PBS followed by an overnight incubation at 4 °C with 30% sucrose in PBS, in order to preserve tissue architecture after cryopreservation. Tumors were then embedded in optimal cutting temperature compound (OCT) (Electron Microscopy Sciences, Hatfield, USA) and stored at −80 °C. Cryosections of 8 µm thick were generated on a LEICA CM3050 cryostat. Tumors cryosections were analyzed by immunofluorescence to detect murine endothelial cells thanks to a specific endothelial tight junction CD-31 (also named PECAM1) and nuclei with Hoechst. Briefly, the slides were rinsed with demineralized water to remove OCT, saturated with MaxBlock Blocking (Active Motif, La Hulpe, Belgium), rinsed and then exposed to anti-CD-31 purified Rat Anti-Mouse primary antibody (BD, Franklin Lakes, USA) at 1/400 in Dako antibody diluent (Dako, Glostrup, Denmark), at 4 °C, overnight. The slides were then rinsed and incubated at room temperature for 30 min with a secondary antibody coupled to Alexa Fluor 594 Chicken Anti-Rat IgG (emission in the red wavelengths, from Thermo Fisher Scientific, Waltham, USA) and DNA intercalant Hoechst at 1/500 in Dako antibody diluent (Dako, Glostrup, Denmark) to counterstain nuclei in blue. Slides were, afterward, rinsed with PBS and demineralized water, and a cover-glass was mounted using Dako mounting medium (Dako, Glostrup, Denmark). Observations were carried out with the Fluoview FV1000 confocal microscope. Eventually, red fluorescence (594 nm excitation) corresponds to CD-31 of murine endothelial cells, green fluorescence (488 nm excitation) to DiO, and blue (450 nm excitation) to nuclei.

### Statistics

Triplicates were collected for each *in vitro* experiment, each experiment being carried out independently at least three times. The data set was assessed using Fisher and Student’s *t*-test (bilateral and difference in variances determined by Fisher test). All data were expressed as mean ± standard deviation, and overall statistical significance was set at *p* < .05.

## Results

### Micelle and polymersome nanocarriers characterizations

**Figure 1.**
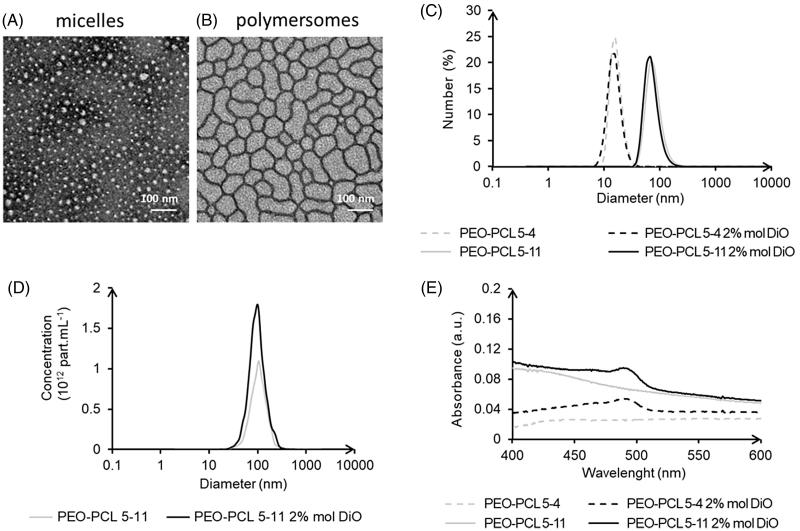
Micelles (PEO-PCL 5-4) and polymersomes (PEO-PCL 5-11) size and charge characterizations: Transmission electron microscopy (A,B); DLS analyses (C); nanoparticle tracking analyses (D); and UV-vis analyses (E).

Both PEO-PCL 5-4 micelles or PEO-PCL 5-11 polymersomes exhibited a spherical shape (Figure 1(A,B)), with larger dimensions for polymersomes as confirmed by hydrodynamic diameter measurements using DLS (Figure 1(C) and Table 1). Further diameter analyses were conducted using Nanoparticle Tracking Analysis (NTA) for polymersomes only, as micelles were under the limit of detection of the NTA device (Figure 1(D)). The morphology of these vesicles was thoroughly characterized in a previous study (Till et al., 2014), and further measurements (SLS/DLS characterization, Field Flow Fractionation, SANS, and cryo-TEM) confirmed these findings (supplementary data, Figure S2–S4). No significant change in diameter was observed between empty and DiO-charged nanocarriers. Zeta potentials analyses showed slightly negative surface charges (Table 1), and UV-vis measurements revealed a net encapsulation of the DiO with a single peak around 490 nm (Figure 1(E)). The UV-vis signal and diameter stability were examined at room temperature over 4 or 9 days, respectively (Supplementary data Figure S5). The hydrodynamic diameters and DiO specific peak area or height remained constant, which confirmed that the nanocarriers can be stored for a few days at room temperature. To examine possible leakage of the DiO probe, the DiO release from the nanocarriers diluted at 50 µM polymer (equivalent to 1 µM DiO, as used in the next assays) was assessed at 37 °C, over 24 h, by dialysis in ultrapure water. No significant signal was detected in the external solution. In the dialysis tube, there was no significant decrease of the peak area and of the peak height except for micelles at 24 h (Supplementary dataTable S1). This slight decrease in peak height for micelles was not significantly different from the one showed for the control and could be imputable to the experience itself: decrease of the fluorescence at each UV reading.

**Table 1.** Micelles and polymersomes physicochemical analysis: DLS hydrodynamic diameter (D_H_) in number and intensity average, NTA hydrodynamic diameter, and Zeta potential measurements.

	Empty	Charged with 2% mol DiO
Micelles PEO-PCL 5-4 D_H_ in number average (nm) D_H_ in intensity average (nm) Zeta potential (mV)	14 ± 1 24 ± 0 −13 ± 3	14 ± 2 25 ± 4 −11 ± 4
Polymersomes PEO-PCL 5-11 D_H_ in number average (nm) D_H_ in intensity average (nm) NTA diameter (nm) Zeta potential (mV)	72 ± 6 135 ± 11 111 ± 9 −4 ± 1	65 ± 5 117 ± 10 93 ± 10 −3 ± 1

### Internalization of nanocarriers by human endothelial and tumor cells

#### Internalization of mono-and co-cultures in monolayer

Cytotoxicity was assessed as a preliminary and mandatory step to ensure suitable safety of the systems (Supplementary data Figure S6). After 24 h of exposure, as expected, none of the treatments decreased significantly HUVEC and HCT-116 viability. Live HUVEC and HCT-116 were observed using confocal microscopy after 24 h exposure to nanocarriers charged with DiO (Supplementary data Figure S7). After exposure of HUVEC to micelles, DiO was visible in the cytoplasm. Polymersomes induced a more intense although blurrier fluorescence. A slight fluorescent background is notable for the non-treated HUVEC but discernible from the DiO fluorescence. In the HCT-116 cells with smaller dimensions, the detection of DiO from micelles is less obvious compared to the control. Nonetheless, after exposure to polymersomes, HCT-116 was clearly marked.

**Figure 2.**
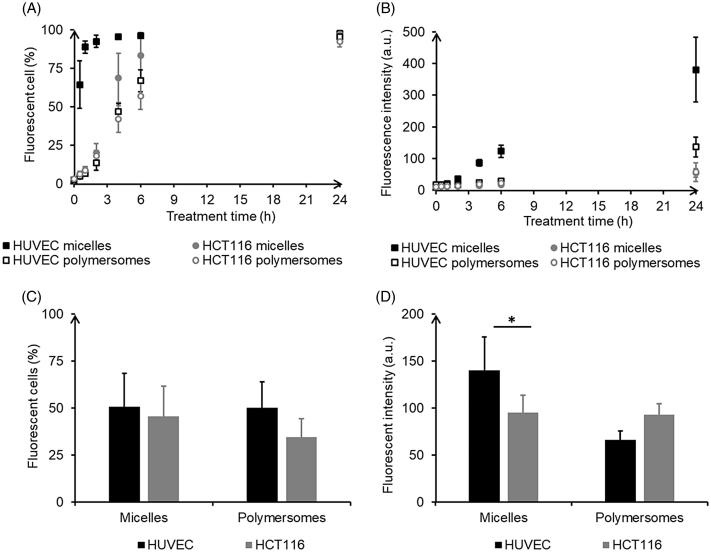
Quantification by flow cytometry of internalization of micelles and polymersomes charged with DiO in HUVEC and HCT-116: kinetics in monoculture (A,B) and 24 h internalization in co-culture (C,D).

Flow cytometry confirmed DiO internalization using HUVEC, and HCT-116 cells treated with either micelles or polymersomes. A more efficient delivery of DiO by micelles was observed in HUVEC. Both the proportion of cells labeled with DiO (Figure 2(A)) and the intensity of DiO fluorescence in those cells (Figure 2(B)) increased with time. HUVEC treated with micelles exhibited the fastest internalization process with more than 60% labeled cells after only 0.5 h of exposure and a plateau around 95% reached after 4 h. The three other conditions led to a similar kinetic in three steps: first, a slow increase of the internalization rate; second, after 2 h a crucial acceleration until eventually a plateau was reached. The proportion of labeled cells at the plateau is equivalent for HUVEC or HCT-116 exposed to micelles (around 97%), whereas slightly underneath for cells exposed to polymersomes (95% and 92%, respectively). HUVEC treated with micelles also showed the highest intensity of fluorescence, whereas those treated with polymersomes exhibited a lower intensity of fluorescence but still higher than those for HCT-116 treated with micelles or polymersomes. In the co-culture assay, the tendency for HUVEC to integrate more DiO, especially, from micelles was kept: slightly more HUVEC than HCT-116 were labeled for both nanocarriers (Figure 2(C)) and micelles induced significantly more fluorescence intensity in labeled HUVEC than in HCT-116 (Figure 2(D)). However, there was no real specificity of the nanocarriers as the proportion of cells of each type labeled with DiO was not significantly different when exposed to micelles (51% of HUVEC and 46% of HCT-116) or polymersomes (50% and 34%).

#### DiO distribution in 3D tumor spheroids

**Figure 3.**
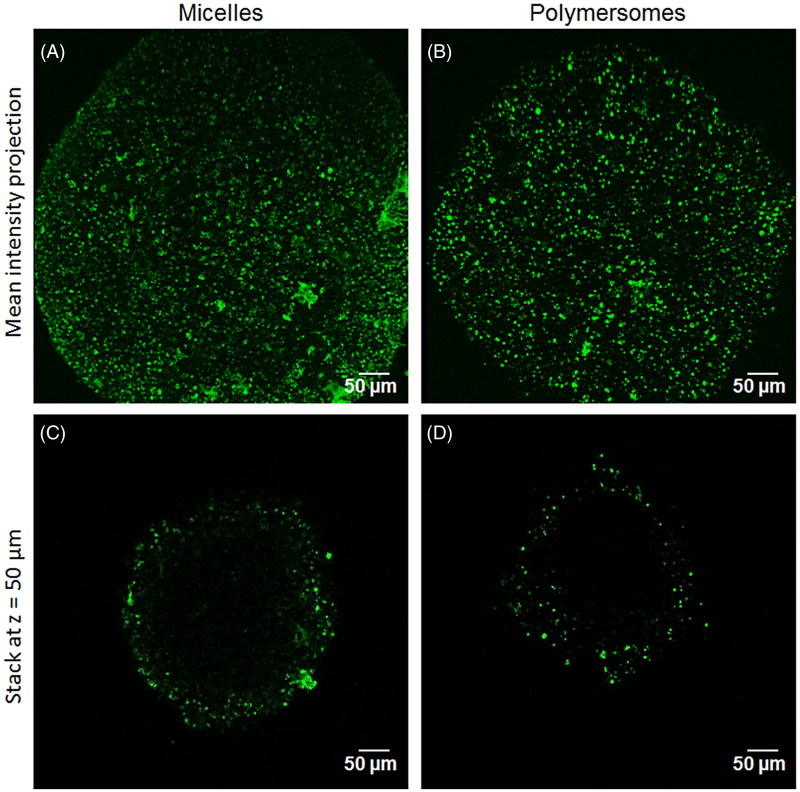
DiO penetration in 3D tumor spheroid depending on the nanocarriers: two-photon microscopy images of the mean intensity projection of DiO fluorescence from HCT-116 spheroids exposed for 24 h to micelles (A) and polymersomes (B). C and D are optical sections at *z* = 50 µm depth of the spheroid A and B, respectively.

The internalization capacity of DiO from the nanocarriers, proven in conventional 2D culture, was then tested in 3D tumor spheroids. Figure 3 displays two-photon microscopy images of the spheroids treated with nanocarriers for 24 h. DiO internalization by the first cell layers of the spheroid was clearly observable for either micelle (Figure 3(A)) or polymersomes (Figure 3(B)). The penetration capacity was measured on an optical section 50 µm depth from the spheroid surface (Figure 3(C,D)). DiO from micelles penetrated significantly further than DiO from polymersomes (85 ± 35 µm and 68 ± 29 µm, respectively). Depending on their characteristics, the two nanocarriers were thus able to deliver their charge in the first 5–10 cell layers of a tumor spheroid in 24 h, which is crucial for the delivery of therapeutic agents on site. These results mean that once the endothelium barrier is crossed, micelles will tend to be more widely spread within surrounding tumor tissue.

### Nanocarriers translocation across the endothelium barrier

#### Passage of nanocarriers through the endothelium model

**Figure 4.**
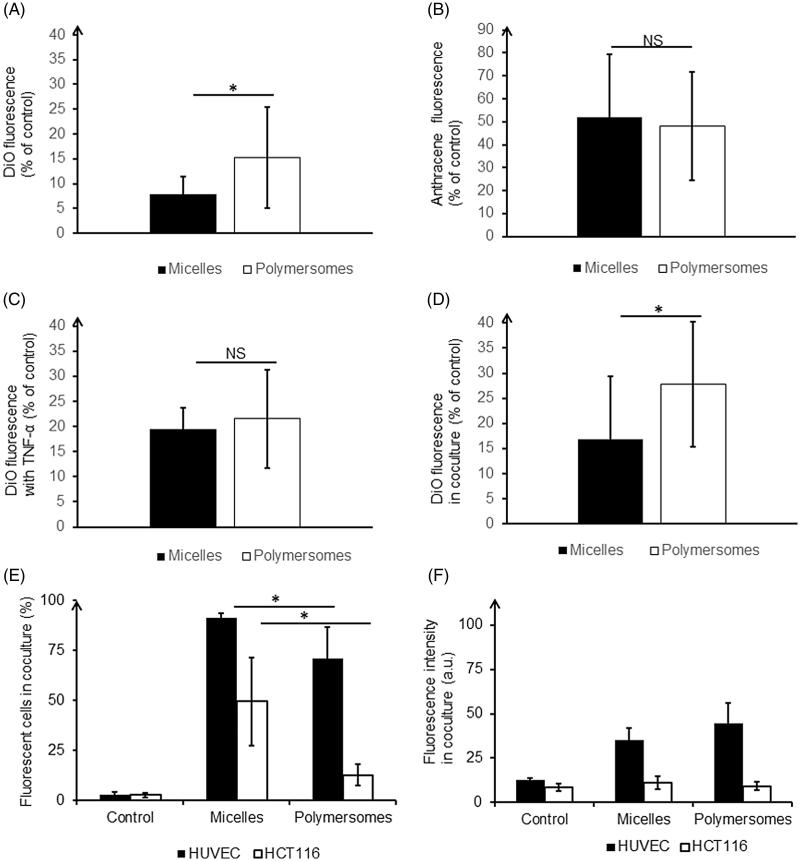
Quantification of nanocarriers’ passage across the endothelium barrier after 24 h of incubation: DiO fluorescence in the receptor chamber (A), Anthracene fluorescence in the receptor chamber (B), DiO fluorescence in the receptor chamber in pro-inflammatory condition (C), DiO fluorescence in the receptor chamber in presence of tumor HCT-116 (D). Intracellular quantification of nanocarriers by flow cytometry: proportion of labeled cells (E), intensity of DiO fluorescence in labeled cells (F). *p < .05. NS: non-significant difference. For each nanocarrier, the DiO fluorescence was significantly lower in the healthy condition compared to the disturb conditions (with TNF-α or in coculture).

After 48 h of the HUVEC monolayer growth and the production of a cohesive endothelium was observed (as shown in Supplementary Figure S8 and confirmed using TEER measurements in Figure S9(A)), nanocarriers were introduced in the donor chamber for a 24 h incubation. Figure 4(A) shows a significant difference in the DiO fluorescence level found in the receptor chamber for micelles and polymersomes. DiO encapsulated in micelles crossed the endothelium almost twice less than DiO encapsulated in polymersomes (8% and 15%, respectively). We showed in 2D classical monolayer (Figure 2) that DiO encapsulated in micelles was massively trapped inside HUVEC after incubation, this process could explain why micelles are less available for passage across the endothelium and then less detected in the receptor chamber. At this step, it was crucial to determine if DiO crossed the endothelium freely after having been released from the nanocarriers, or if the nanocarriers crossed it along with the DiO. Micelles and polymersomes charged with DiO and labeled with Anthracene covalently linked to the polymer PCL were formulated to determine this. The thorough characterization of these nanocarriers showed that their physicochemical characteristics were similar to those of empty nanocarriers as well as for nanocarriers charged with DiO only (Supplementary Figure S10). The endothelium model experiment was carried out in the same manner as previously. Anthracene fluorescence measurements in the receptor chamber showed that around 50% of the Anthracene crossed the endothelial monolayer for both micelles and polymersomes (52% and 48% respectively) (Figure 4(B)). The difference between the two types of nanocarriers was not significant. It appeared thus that the polymers and the DiO were able to cross the endothelium even if it remained unclear if the nanocarriers were still intact afterward.

In a tumor context endothelium is affected by the microenvironment, thus a pro-inflammatory cytokine known to activate endothelial cells (TNF-α) was added in the receptor chamber to simulate such altered environment. TEER measurements confirmed a disruption of the endothelium barrier (Supplementary Figure S9(B)). After 24 h of incubation with charged nanocarriers, the DiO levels in the receptor chamber were higher than in healthy condition, especially for micelles, which smoothed the difference between them and polymersomes (Figure 4(C)). To mimic more closely cells interactions as observed in the micro-environment *in vivo*, HCT-116 tumor cells were seeded in the bottom of the receptor well in co-culture. Approximately two-fold higher levels of DiO were found in the receptor chamber, indicating a significant effect of soluble factors released by tumor cells on endothelium integrity (Figure 4D). Those results underlined the alteration of the endothelium barrier function in pathological conditions. Additionally, flow cytometry experiments were performed after cell detachment to give an indication of the amount of DiO retained in the model of endothelium layer and tumor (Figure 4(E,F)). DiO from micelles labeled a significantly higher amount of HUVEC but at intensities lower than DiO from polymersomes. A significantly higher proportion of HCT-116 was labeled with DiO from micelles rather than from polymersomes (50% and 13%, respectively). Additionally, the intensity of HCT-116 labeling from polymersomes was very low and not significantly different from the control. Thus, even if polymersomes crossed more easily the endothelial barrier, hence more DiO is available in receptor chamber, tumor cells were not prompt to integrate it, contrary to DiO from micelles.

### In vivo *early results*

**Figure 5.**
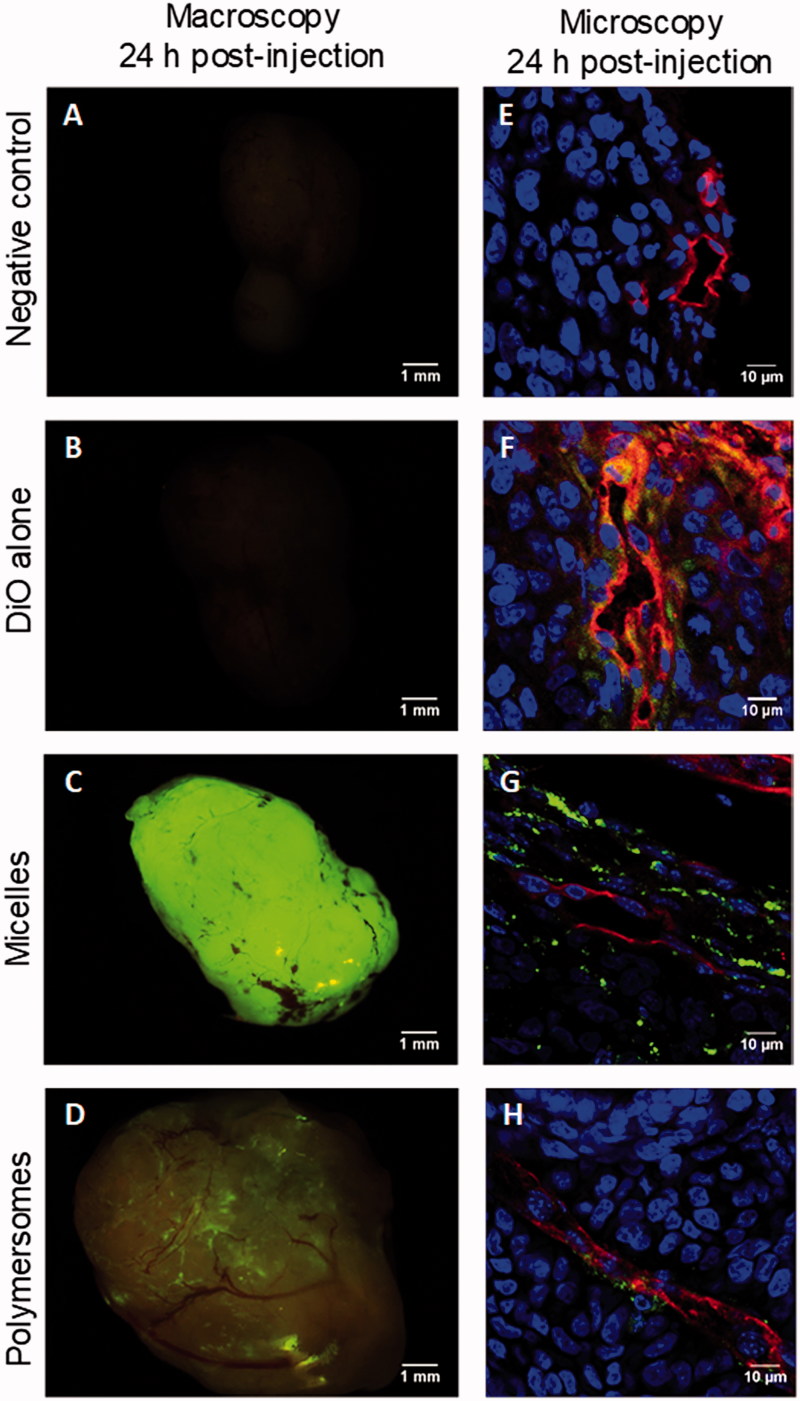
Macroscopic and microscopic (cryosection) fluorescent pictures of explanted HCT-116 tumors 24 h after retro-orbital injection in mice of 100 µL of PBS (A,E), DiO alone (B,F), micelles charged with 20% mol DiO (C,G), or polymersomes charged with 20% mol DiO (D,H). DiO fluorescence (green), and in cryosection nuclei stained with Hoechst (blue), and CD-31+ murine endothelial cells detected by immunofluorescence (red).

A preliminary *in vivo* study was carried out to confirm the *in vitro* results. Nanocarriers charged with 20% mol DiO were formulated, especially for the *in vivo* experiments. Their physicochemical characteristics were comparable to nanocarriers with 2% mol DiO except for their UV-vis signal (Supplementary Figure S11). Right after retro-orbital injections, no specific fluorescence was seen through the skin, apart from the skin autofluorescence itself. Twenty-four hours after injection, the mice were sacrificed, the main storage organs, lungs, livers, and kidneys, were observed (Supplementary data Figure S12), but a focus was made on explanted tumors. In the mouse treated with DiO alone, the tumor showed no fluorescence discernable from the controls (Figure 5(A,B)). The tumor from the mouse treated with micelles displayed a very intense fluorescence (Figure 5(C)) and the one treated with polymersomes a lower but still distinct fluorescence (Figure 5(D)). Interestingly, although fluorescence signal from micelles was homogeneously spread all over the tumor, the one from polymersomes seemed to closely follow the vasculature architecture. Further analyses of the tumors were conducted using confocal microscopic observations of cryo-sections of each tumor after an immunolabelling against CD-31, a protein specific of endothelial cells constituting the endothelium in blood vessels (Figure 5(E–H)). As for macroscopic observations, cryo-sections associated with micelles exhibited the highest DiO fluorescence signal. DiO fluorescence from micelles was located in the cytoplasm of the cells up to tens of micrometers from the blood vessels and did not seem to be specifically associated with endothelial cells. On the contrary, DiO fluorescence from polymersomes was mainly detected close to cells positively labeled for CD-31. The tumor of the mouse treated with DiO alone led to a cryosection with a diffuse DiO fluorescence that was not visible at a macroscopic scale. Overall, it suggested that DiO alone can reach the tumor but in a largely less specific and efficient way than DiO encapsulated in nanocarriers. Moreover, it confirms that DiO from micelles diffuse easily in the tumor, whereas polymersomes were confined in its vicinity despite their enhanced capacity to cross the endothelium.

## Discussion

Previously, the two polymeric nanocarriers studied here, PEO-PCL 5-4 micelles and PEO-PCL 5-11 polymersomes, have successfully shown their capacity to deliver Pheophorbide A for anti-cancer photodynamic therapy (Knop et al., 2009; Gibot et al., 2014; Till et al., 2016b). Equivalent elongated self-assemblies (PEO-PCL 2-7) did not show any advantages in terms of efficacy in these former studies, thus the focus was made on spherical micelles and polymersomes for further studies on such systems. In this work, we took a step back and focused on the path of the nanocarriers and its encapsulated drug from the blood vessel to the tumor across the endothelium. Two main questions were to answer. First, do nanocarriers interact with endothelial cells from the blood vessel, and if so, is there a specificity in the interaction with endothelial or cancer cells? Second, are nanocarriers able to cross an endothelial monolayer in healthy conditions or in the tumor region, if so at which proportion?

The first question was assessed by studying the *in vitro* internalization of PEO-PCL micelles and polymersomes in human primary endothelial cells HUVEC and colorectal HCT-116 tumor cells. It was demonstrated that DiO was internalized by both cell types in 2D cell culture and in 3D HCT-116 tumor spheroids. A previous study from our laboratories suggested that antitumor drug release occurred by direct jump from the nanocarriers PEO-PCL micelles (PEO-PCL 2-2.6 and 5-4) to the human colon cancer cells (HCT-116) (Till et al., 2016a). On the other hand, the review of Christian et al. (2009) concluded that the main release mechanism of encapsulated agents from PEO-PCL polymersomes is through PCL hydrolytic degradation, which is accelerated intracellularly in endolysosomes, thus after internalization of the nanocarriers. It has also been shown that endocytosis played a crucial role in the internalization of other PEO-based micelles (Zhang et al., 2016). As confirmed by the co-culture experiments in monolayer, DiO signal did not seem to be cell type specific. Small differences were nonetheless observed, in kinetics, intensity, and proportion of labeled cells, more important for HUVEC. These differences were also linked to the nanocarriers type. DiO signal was faster and more intense for micelles compared to polymersomes. It was observed, especially, in HUVEC, but the trend was still present in HCT-116. DiO from micelles were also internalized deeper into HCT-116 tumor spheroids than polymersomes. If we consider the possible internalization of the nanocarrier itself and not just the encapsulated compound, these results were not surprising. Size has indeed been widely documented as a factor influencing cell internalization of nanomaterials (Massignani et al., 2009; Chang et al., 2014; Ferrari et al., 2014). The nanocarriers of our study presented indeed very dissimilar diameters for a same surface chemistry, size is thus thought to be the most influent parameter here. However, Zhang et al. (2013) suggested that at equal size, the internalization of copolymer nanocarriers in HUVEC and tumor cells is driven by the hydrophilic-hydrophobic ratio. The PEO-PCL 5-5 micelles of that study, which are the closest from our PEO-PCL 5-4 micelles, exhibited, as found here, the highest uptake efficiency in different cell lines. Other micelles, closer in size to our PEO-PCL 5-11 polymersomes, with low hydrophilic-hydrophobic ratio, showed also lower uptake efficiency. Copolymers ratio resulting in different nanocarrier’s size and surface properties could influence the internalization of endothelial and cancer cells, without inducing specificity for one or the other type of cells.

The second part of this work gave evidence that nanocarriers could cross an endothelium model, and at higher proportions when endothelial cells were activated with TNFα or in distressed environments compared to a healthy one. Approximately, 17% of polymeric nanocarriers could cross a confluent HUVEC monolayer in healthy conditions. After disruptions of the HUVEC monolayer by TNF-α or co-culture with tumor HCT-116 in the receptor well, up to 28% of the nanocarriers could cross it. It is known that in general health condition, the passage should be very restricted with nanocarriers as only transcellular passage should be possible with diameter higher than 15 nm (Komarova & Malik, 2010). However, such translocation of some nanomaterials across a healthy monolayer of endothelial cells was already seen in the literature. Jayagopal et al. (2008) observed translocation of solid lipid nanoparticles through endothelial cells from bovine aortas. Zhao and Lin worked on endothelial layers from blood-brain barrier, for Zhao, there was a possible translocation of the nanoparticles, or for Lin, at least of the nanocarriers charge (Lin et al., 2016; Zhao et al., 2016). The increase in DiO’s passage induced by a cytokine or cancer cells was more expected. Both para- and transcellular pathway are triggered by external stimuli, and the presence of cancerous cells or disturbing agent can increase the transport rate and contribute to the creation of a fenestrated discontinuous endothelium (Dvorak et al., 1988; Baban & Seymour, 1998; Maeda, 2001). Interestingly, DiO intensity in HUVEC quantified using flow cytometry was lower when HCT-116 was seeded on the receptor cells. This could mean that HCT-116 liberates soluble factors or extracellular vesicles reducing the uptake of HUVEC and inducing the higher permeability in this co-culture model. Baban & Seymour (1998) suggested indeed that vascular permeability was regulated by tumor-secreted growth factors notably vascular endothelial growth factor (VEGF), a cytokine is known to be expressed by HCT-116 cells (Ahluwalia et al., 2013). Furthermore, both in healthy conditions and in co-culture condition with tumor cells, the proportion of DiO crossing the endothelium was significantly lower for micelles compared to polymersomes. Despite that, HCT-116 on the bottom well was still more labeled with DiO from micelles than polymersomes. It indicates that even if less DiO from micelles were available in the bottom well, it was more prompt to be internalized by cancer cells. These results are in accordance with 2D and 3D observations led on 3D tumor spheroids where DiO from micelles was able to deeper penetrate in tumor spheroids than DiO from polymersomes. Further enhancement of the tumor-like environment could be made to keep investigating this. A longer pre-culture of the endothelial cells on the inserts with 3D spheroids in the lower chamber could be set up, with an exhaustive investigation of the factors released by tumor cells. A model of blood vessel wall including a layer of endothelial cells and several layers of muscular cells was developed by Chetprayoon et al. (2015, 2016) and could be another step toward a complete 3D blood vessel *in vitro* produced by tissue engineering.

To answer the question of the integrity of the nanocarriers after crossing the endothelium, Anthracene covalently linked with PCL was used as a label for the nanocarriers in the second part of this study. However, its detection using confocal microscopy or flow cytometry was not technically feasible. A Fluorescence Resonance Energy Transfer (FRET) experiment was considered to answer this question, unfortunately, commercial availability of labeled PCL is very limited and no correct pair of donor and acceptor could be found. Theoretically, this couple—composed of encapsulated DiO and another fluorophore covalently linked to the polymers—would have expressed a high specific signal when in close vicinity (1–10 nm typically). This signal should drastically fall when the distance increases, thus indicating that the DiO is not in close vicinity of the polymer anymore; hence, it was released from the nanocarrier. But, we still confirmed that Anthracene covalently linked to polymers had indeed crossed endothelial monolayer because it was found in the lower chamber of the endothelium model. However, as previously mentioned, it was not technically feasible to discern whether the nanocarriers were intact and still encapsulating the DiO.

Early *in vivo* results validated the endothelium transwell model by confirming our previous findings. Nanocarriers charged with 20% mol DiO were formulated for this experiment, with a fluorescence signal interestingly lower for polymersomes than for micelles. DiO self-quenching could be the issue here (Heberle et al., 2005; Buboltz et al., 2007; Zhao et al., 2007; Chen et al., 2008). However, it was not seen for micelles although their core is expected to be narrower than polymersome inner space. This could also be due to a lower encapsulation capacity in the polymersomes despite their larger diameter compared to micelles and the general literature allegation (Balasubramanian et al., 2016). Alibolandi et al. (2016) suggested, notably, that PEO in the confined inner space of a polymersome induces a very crowded environment that decreases its encapsulation capacity. Nonetheless, the DiO level was sufficient to detect it after injection in mice, allowing the *in vivo* experiment to be carried out, but restraining comparison of DiO intensity from micelles to polymersomes. DiO was observed in the lungs, liver (especially for free DiO and DiO from micelles), and kidney, but it was effectively more uptaken by tumor when encapsulated in nanocarriers than as a free molecule. In cryosections, it was seen that DiO from micelles diffused largely in tumor cells, far from the vasculature, whereas DiO from polymersomes stayed in the vicinity of blood vessels. It matched with the hypothesis that micelles internalized faster in a large number of cells, whereas polymersomes which crossed the endothelium at higher amount are more slowly internalized in cancer cells. A development of this *in vivo* confirmation could be made by looking at the rate of internalization and washing from the tumor as proposed in Ke’s work (Ke et al., 2017, 2018).

Overall, we have seen that the polymeric nanocarriers were internalized by endothelial and cancer cells, without specificity in this interaction. Furthermore, around 17% of nanocarriers could cross the endothelium model used herein healthy condition. This proportion was increased in distressed environment of up to 28%. Nonetheless, important losses of encapsulated compounds can be predicted, induced by all nonspecific interactions with the endothelium and translocation through the blood vessels in healthy area. This has to be taken into account when designing a new nanocarrier for nanomedicine, especially considering that these losses seemed to be dependent on some physicochemical properties of the nanocarrier, such as its size. In a radical change of paradigm, endothelial cells in non-cancerous conditions could be considered as a good target for those nanocarriers, especially for polymersomes. But for more traditional anti-cancer therapy, other strategies such as active-targeting of the nanocarriers should be further looked into in order to enhance cell specificity (Peer et al., 2007; Guo et al., 2016; Ke et al., 2018).

## Conclusions

Nanocarriers, PEO-PCL 5-4 micelles and PEO-PCL 5-11 polymersomes, presented the ability to cross the endothelium barrier to reach tumor cells, but drug losses are expected throughout this process. Internalization of the encapsulated DiO was confirmed both in endothelial HUVEC cells and tumor HCT-116 cells with no ostensible specificity. Translocation through the endothelium model also occurred in healthy condition. It was nonetheless enhanced (from 17% up to 28%) with the addition of a disturbing agent (TNF-α or cancer cells) as suggested by the EPR theory. The two studied polymeric nanocarriers shared the same surface chemistry but were largely different in size. DiO internalization was faster and more efficient with micelles than polymersomes, especially in endothelial cells. This privileged internalization could induce retention and explain the lower rate of DiO from micelles crossing the endothelium model. Despite a lower translocation capacity, DiO from micelles was, however, once again more internalized in the cancer cells seeded underneath the endothelial monolayer than DiO from polymersomes. The *in vivo* early results eventually confirmed this distinct behavior between micelles and polymersomes. Finally, it seemed that micelles were more efficient drug delivery systems than polymersomes but losses regarding nonspecific internalization and translocation through the blood vessel have yet to be taken into consideration. The on-going development of *in vitro* endothelium and capillaries models could benefit future investigations on the impact of the nanocarriers’ physicochemical characteristics, aiming for an improved ratio of administrated versus delivered dose.
